# Effects of sequencing platforms on the profiling of root mycorrhizal communities in *Pinus densiflora*

**DOI:** 10.71150/jm.2509008

**Published:** 2026-01-31

**Authors:** Ki Hyeong Park, Seung-Yoon Oh, Shinnam Yoo, Yoonhee Cho, Ji Seon Kim, Chang Wan Seo, Chang Sun Kim, Young Woon Lim

**Affiliations:** 1 School of Biological Sciences and Institute of Biodiversity, Seoul National University, Seoul 08826, Republic of Korea; 2 Forest Entomology and Pathology Division, National Institute of Forest Science, Seoul 02455, Republic of Korea; 3 School of Advanced Biosciences, Changwon National University, Changwon 51140, Republic of Korea; 4 Department of Forest Biomaterials and Technology, Swedish University of Agricultural Sciences, Uppsala 75007, Sweden; 5 Forest Biodiversity Division, Korea National Arboretum, Pocheon 11186, Republic of Korea

**Keywords:** mycorrhizal fungi, mycobiome, *Pinus densiflora*, Illumina MiSeq, PacBio Sequel

## Abstract

Next-generation sequencing (NGS) has become a powerful and efficient tool for surveying mycorrhizal mycobiome diversity, surpassing classical methods in accuracy and throughput. Long-read NGS techniques are increasingly applied under the assumption that they provide better taxonomic resolution, yet their use often lacks a balanced evaluation against the established strengths and limitations of widely used short-read NGS technologies. This study compares Illumina MiSeq and PacBio Sequel platforms in analyzing the mycorrhizal mycobiome of *Pinus densiflora* roots, focusing on how sequencing platforms and database choice influence taxonomic resolution and diversity patterns. Both platforms detected mycorrhizal taxa with similar taxonomic resolution, recovering nearly all taxa previously reported from pine roots. Most mycorrhizal taxa were shared between datasets, although several taxa were detected exclusively by one platform. In terms of diversity, the short-read dataset showed higher diversity due to greater sequencing depth, whereas the long-read dataset offered improved identification of rare or closely related taxa owing to longer sequence information. Moreover, supplementing reference databases with locally derived sequences enhanced taxonomic resolution and the detection of native taxa in both approaches, with a stronger effect for the long-read dataset. Overall, our results emphasize that short- and long-read sequencing each have distinct advantages for mycorrhizal community analysis, and that the use of curated local reference databases is essential to maximize taxonomic resolution and improve the detection of regionally unique taxa.

## Introduction

Mycorrhizal fungi form symbiotic associations with plant roots and play essential roles in forest ecosystems, particularly in nutrient cycling (Smith and Read, 2008). The most geographically widespread mycorrhizal types are ectomycorrhiza (EM), arbuscular mycorrhiza (AM), and ericoid mycorrhiza (ErM), which altogether colonize more than 80% of terrestrial plants (Brundrett, 2009). Traditional methods used to study these fungi, such as fruiting body collection, strain isolation, or root-tip sequencing, offer foundational insights but face limitations due to the ephemeral nature of fruiting bodies, the labor-intensive process, and the inability to detect species with restricted distributions.

Next-generation sequencing (NGS) has transformed mycorrhizal research by allowing comprehensive community profiling with reduced effort and minimal environmental disturbance (Ruppert et al., 2019). Among NGS platforms, Illumina MiSeq and HiSeq are widely utilized for sequencing internal transcribed spacer (ITS) subregions such as ITS1 or ITS2, providing sufficient depth for ecological surveys (Nilsson et al., 2019). However, short-read NGS platforms capture only partial ITS region, and the optimal subregion for species level identification differs among fungal taxa (Tedersoo et al., 2021). Furthermore, distinguishing closely related species is difficult with short-read sequencing in certain fungal groups, as they exhibit high sequence similarities in the ITS subregions. To overcome these limitations, long-read technologies, such as the Pacific Biosciences (PacBio) platform, have been employed to sequence the full-length ITS region, which improves taxonomic discrimination by capturing more informative sites (Tedersoo et al., 2018; Walder et al., 2017). Nevertheless, long-read sequencing platforms are substantially more expensive to achieve the same sequencing throughput (Kennedy et al., 2018; Tedersoo et al., 2018).

*Pinus densiflora* Sieb. et Zucc. (red pine) is a well-known ectomycorrhizal tree, which forms obligatory relationship with EM fungi (Policelli et al., 2020). It is distributed mainly in temperate forests of Asia, including Japan, northeastern China, and eastern Russia. It is also the predominant tree species in South Korea, accounting for 30% of the entire tree population (Choung et al., 2020). Mycorrhizal fungi from the pine roots in South Korea have been studied through various DNA sequencing methods (Cho et al., 2021; Park et al., 2020; Sim and Eom, 2009; Yoo et al., 2022). Our previous study using Illumina MiSeq recently revealed distinct differences between the mycorrhizal communities of *P. densiflora* roots and surrounding soil (Park et al., 2021). These previous results showed that many mycorrhizal species detected by sequencing could not be identified because their reference sequences were missing from public databases. This gap exists due to the understudied and region-specific nature of these fungi.

Despite the increasing use of long-read sequencing platforms, short-read NGS methods still dominate due to their cost-effectiveness and well-established protocols. The advantages and limitations of each sequencing platform, as well as practical considerations for optimization, have been thoroughly discussed (Castaño et al., 2020). However, only a limited number of studies have explicitly compared their performance in characterizing microbial communities using real-world data (Furneaux et al., 2021; Hu et al., 2022). In this study, we compared fungal mycobiome results from *P. densiflora* root samples across South Korea using two sequencing platforms (PacBio Sequel and Illumina MiSeq). We aimed to assess the influence of sequencing platforms on analyzing mycorrhizal community by comparing taxonomic assignment and diversity. Furthermore, we analyzed importance of including local sequences to database in taxonomic assignment. Our findings provide insights into the strengths and limitations of each platform for mycorrhizal community analysis and offer practical guidance for future studies using NGS-based fungal barcoding.

## Materials and Methods

### Sample collection, PCR amplification, and sequencing

We used samples from a previous study that profiled the mycobiome of *P. densiflora* roots using Illumina MiSeq (Park et al., 2021). Between 2019 and 2020, root samples were collected from 80 trees across 16 forests dominated by *P. densiflora* (Table S1). At each site, five healthy mature trees were randomly selected. Two lateral root segments were obtained from opposite directions of each tree using sterilized scissors. Root samples were sterilized and prepared as described in a previous study (Park et al., 2021). We removed the soil and root tissues of other plants from the root samples by gently washing them in distilled water. After washing, all fine roots with fresh-looking root tips were separated with sterilized scissors. Fine root samples were sterilized by submerging in 3% hydrogen peroxide for 1 min, then rinsed three times with sterile distilled water to remove contaminants. After sterilization, we air-dried the root samples on sterilized filter paper placed on a clean bench overnight. Dried roots were ground with liquid nitrogen using a sterilized mortar and pestle. DNA was extracted using the MoBio PowerSoil DNA Isolation Kit (MoBio Laboratories, USA).

We used the ITS2 region MiSeq dataset generated in Park et al. (2021) for further sequence processing. For the PacBio dataset, PCR was conducted following the PacBio Barcoded Universal Primers for Multiplexing Amplicons protocol (https://www.pacb.com/wp-content/uploads/Procedure-Checklist-Preparing-SMRTbell-Libraries-using-PacBio-Barcoded-Universal-Primers-for-Multiplexing-Amplicons.pdf). ITSOF-T and LR5F primers were used to amplify the entire ITS region (Nilsson et al., 2019; Taylor and McCormick, 2008; Tedersoo et al., 2008). PCR was performed in triplicate using AccuPower PCR PreMix (Bioneer, Korea). The first-round PCR conditions were 95°C for 10 min, 20 cycles of 95°C for 1 min, 55°C for 1 min, and 72°C for 2 min, followed by a final extension at 72°C for 10 min. PCR products were checked on 1% agarose gel and purified using the Expin^TM^ PCR SV kit (GeneAll Biotechnology, Korea). In the second round of PCR, PacBio barcodes were attached using PacBio barcode primers (Pacific Biosciences, USA) following the same PCR protocol. After purification, concentrations of the amplicon libraries were measured using NanoDrop^TM^ 2000 (Thermo Fisher Scientific, USA) and pooled in equimolar quantities. Downstream processes and amplicon sequencing were conducted at Macrogen (Korea) using the PacBio Sequel platform on four Single Molecule Real Time (SMRT) cells (Pacific Biosciences, USA). The amplicon sequencing data are available from the NCBI Sequence Read Archive (PRJNA894948 for PacBio and PRJNA703822 for MiSeq).

### Sequence processing and taxonomic assignment

Among the 80 samples, data for two samples (GW02P-4, GW04P-3) were discarded from each dataset due to insufficient sequence reads inspected by rarefaction curves. For the PacBio dataset, demultiplexing and Circular Consensus Sequences (CCS) generation were conducted at Macrogen (Korea). CCS sequences were processed using QIIME v.1.8.0 (Caporaso et al., 2010), and subjected to quality filtering (Q ≥ 30). The full ITS region of the sequences was extracted using ITSx v.1.1b (Bengtsson-Palme et al., 2013), and sequences shorter than 300 bp were filtered. The operational taxonomic units (OTUs) were clustered with a 99% similarity model using the open-source sequence search tool VSEARCH v. 2.6.2 (Rognes et al., 2016). Chimeric sequences were filtered using the ‘uchime_denovo’ methods. The post-clustering curation method LULU (Frøslev et al., 2017) was performed (minimum_match = 99) to merge similar co-occurring OTUs. For the Illumina MiSeq dataset, raw sequences from the previous study were processed using the same methods, skipping CCS generation.

The database for taxonomic UNITE v. 8.2 (Abarenkov et al., 2010) and 3,711 ITS sequences (including 1,261 mycorrhizal sequences) from the Seoul National University Fungus Collection (SFC) appended (hereafter, UNITE+local database). The SFC database, built from locally collected specimens, comprises 1,238 species (including 968 species not present in UNITE v. 8.2). Although the database is not publicly accessible, detailed taxonomic information is provided in Table S2. Taxonomy was assigned with the NCBI BLASTn algorithm for each dataset. For each query, the top 10 hits were examined and only matches with ≥ 70% alignment coverage were considered. Taxa were assigned using a stepwise similarity scheme (97–100% identity for species, 90–97% for genus, lower identities for higher ranks) as following the previous study (Tedersoo et al., 2022). Non-fungal sequences and rare OTUs (< 0.005% of the total number of sequences) were filtered (Bokulich et al., 2013). For both the Miseq and PacBio datasets, FUNGuild (Nguyen et al., 2016) was utilized to sort mycorrhizal taxa, and each taxon was manually checked based on literature and the FungalTraits database (Põlme et al., 2020). In analyses, only the OTUs that were identified at least to the genus level with a reliable reference were categorized into a guild to improve credibility. Unless otherwise stated, all tests were conducted on the mycorrhizal OTUs only.

### Platform effect comparison

The influence of sequencing platforms on the mycobiome was analyzed in terms of taxonomic assignment and diversity. All statistical analyses were performed in R v.4.1.2. (R Core Team 2021). For taxonomic assignment comparison, identification resolution was measured based on the taxonomic rank of each OTU. The number of each ectomycorrhizal taxa across all taxonomic ranks was compared between the MiSeq dataset and the PacBio dataset.

For diversity comparison, the number of mycorrhizal OTUs and sequences was compared using rarefaction curves generated with the vegan R package, using a minimum depth (n = 190) with 100 iterations (Oksanen et al., 2013). Alpha diversity indices (chao1, Shannon) were calculated in QIIME. The reproducibility of the diversity survey using different sequencing methods was compared by calculating the number and relative abundance of taxa. The relationship between the number of OTUs from the two datasets was tested with a cor.test function (based on Spearman correlation) in R. The efficiency of both sequencing methods was evaluated by comparing the relative abundance of unidentified taxa across taxonomic levels (phylum to genus). The dissimilarity between the two datasets was analyzed with permutational multivariate analysis of variance (PERMANOVA) statistical test, using adonis in the vegan R package with Jaccard distance and Bray-Curtis dissimilarity. Procrustes analysis was conducted (Gower, 1975) to compare PCoA plots of MiSeq and PacBio datasets using the protest function in the vegan R package, with 10,000 Monte Carlo iterations.

### Database effect on taxonomic assignment

To evaluate the database effect on taxonomic assignments, the UNITE database was prepared alongside the UNITE+local database. To evaluate whether database influence differs by genetic region, a PacBio-ITS2 dataset was prepared by extracting the ITS2 region from the PacBio dataset using ITSx. Taxonomy was assigned using NCBI BLASTn to four combinations (PacBio, PacBio-ITS2 dataset) × (UNITE, UNITE + local database) and compared. Identification resolution was measured based on the taxonomic rank of each OTU.

## Results

### Comparison of sequencing platforms in taxonomic assignment and diversity

**Fig. 1.**
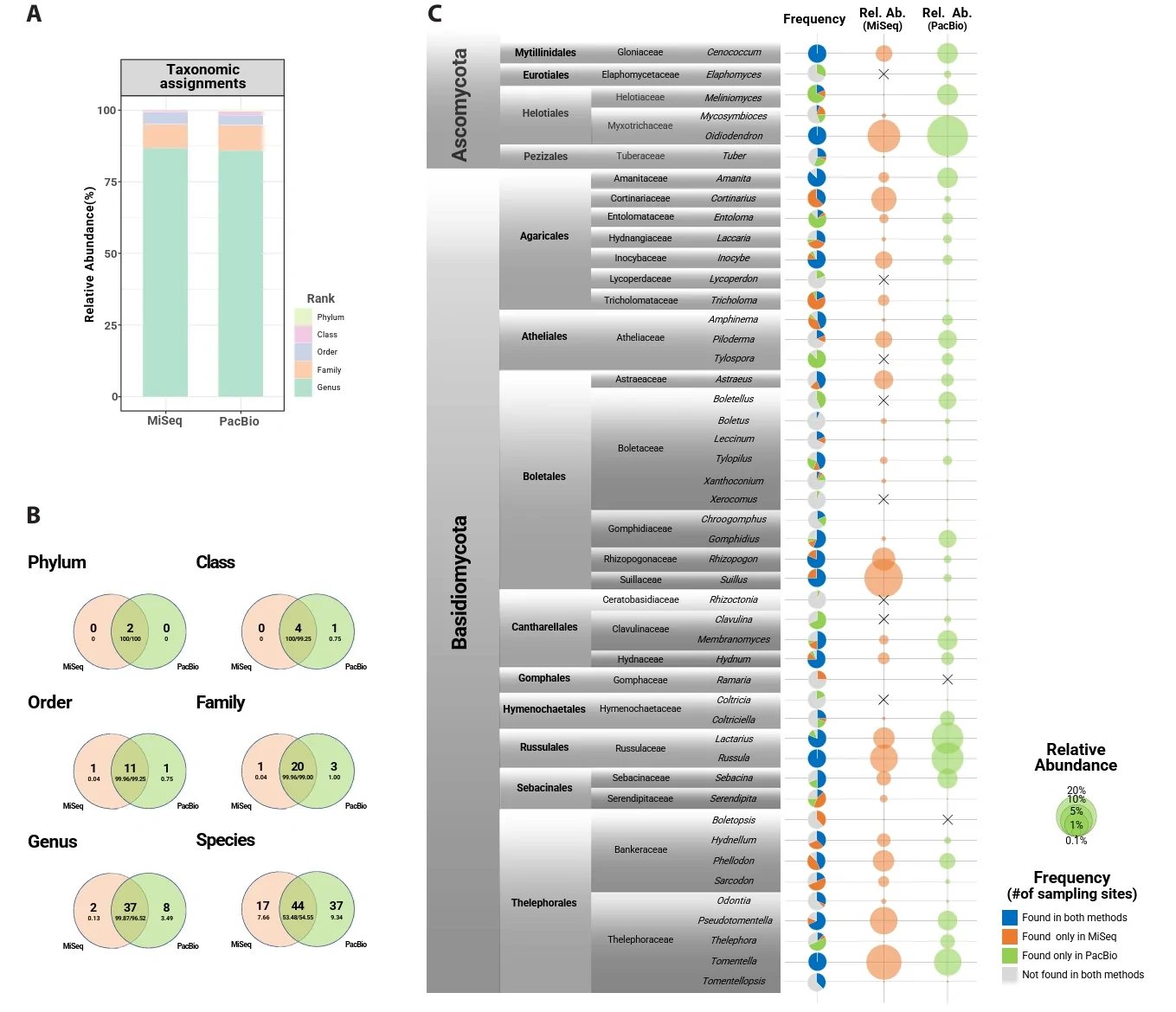
Taxonomic resolution and occurrence comparison of MiSeq (ITS2) and PacBio (full ITS) datasets. (A) The proportion of fungal sequences successfully assigned to each taxonomic level in MiSeq (left) and PacBio (right) datasets. (B) Venn diagrams showing the overlap of the mycorrhizal mycobiome at each taxonomic level (phylum – species) between Miseq and PacBio datasets. The large letters indicate the number of taxa, and small letters represent the relative abundance of those taxa. (C) Frequency (presence/absence; pie chart) and relative abundance of the mycorrhizal fungal genera for MiSeq (orange) and PacBio (green) datasets. The “X” sign in relative abundance indicates the relative abundance is absolutely zero.

From 80 root samples, 3,715,045 MiSeq and 506,675 PacBio sequences were initially obtained. After post-clustering, removing non-fungal sequences and samples with low sequence reads, 2,933,171 sequences and 2,127 OTUs remained in the MiSeq dataset, and 484,280 sequences and 1,109 OTUs in the PacBio dataset. Among them, mycorrhizal fungi accounted for 1,176,839 sequences and 656 OTUs (MiSeq), and 119,551 sequences and 251 OTUs (PacBio). Rarefaction curves approached saturation for most samples in both datasets but did not reach a complete plateau (Fig. S1). The length distribution of OTUs showed a clear difference between the MiSeq and PacBio datasets (Fig. S2). MiSeq OTUs ranged from 315 to 514 bp (mean = 427 bp), whereas PacBio full-length ITS OTUs exhibited a broader range with longer sequences (395-944; mean = 567 bp). On average, 14.17 ± 3.45 genera and 96.73 ± 37.81 OTUs were detected per host tree with MiSeq, while PacBio yielded 13.07 ± 3.44 genera and 31.72 ± 10.27 OTUs per host tree. Less than 15% and 5% of sequences were unassigned at the genus and family levels, respectively, in both datasets (Fig. 1A).

Mycorrhizal community among the total fungal community, two phyla, five classes, 13 orders, 24 families, 47 genera, and 98 species were found across both datasets (Fig. 1B). Forty-four species were shared between datasets, making up over 50% of the total abundance. PacBio detected more exclusive taxa (37 species) than MiSeq (17 species). Basidiomycota was the dominant phylum in both datasets (MiSeq: 86.17%; PacBio: 69.54%). Approximately 61–64% of reads were identified at the species level. On average, *Oidiodendron* was the most abundant genus (MiSeq: 10.71%; PacBio: 22.17%), followed by *Tomentella*, *Suillus*, *Russula*, *Lactarius*, *Pseudotomentella*, and *Cenococcum*. *Tomentella*, *Russula*, and *Cenococcum* were detected at every sampling site in both datasets. *Cortinarius*, *Rhizopogon*, and *Suillus* were found in all sampling sites in the MiSeq dataset, but their abundance and frequency were low in the PacBio dataset. A similar but opposite result was found for *Meliniomyces* (Fig. 1C).

**Fig. 2.**
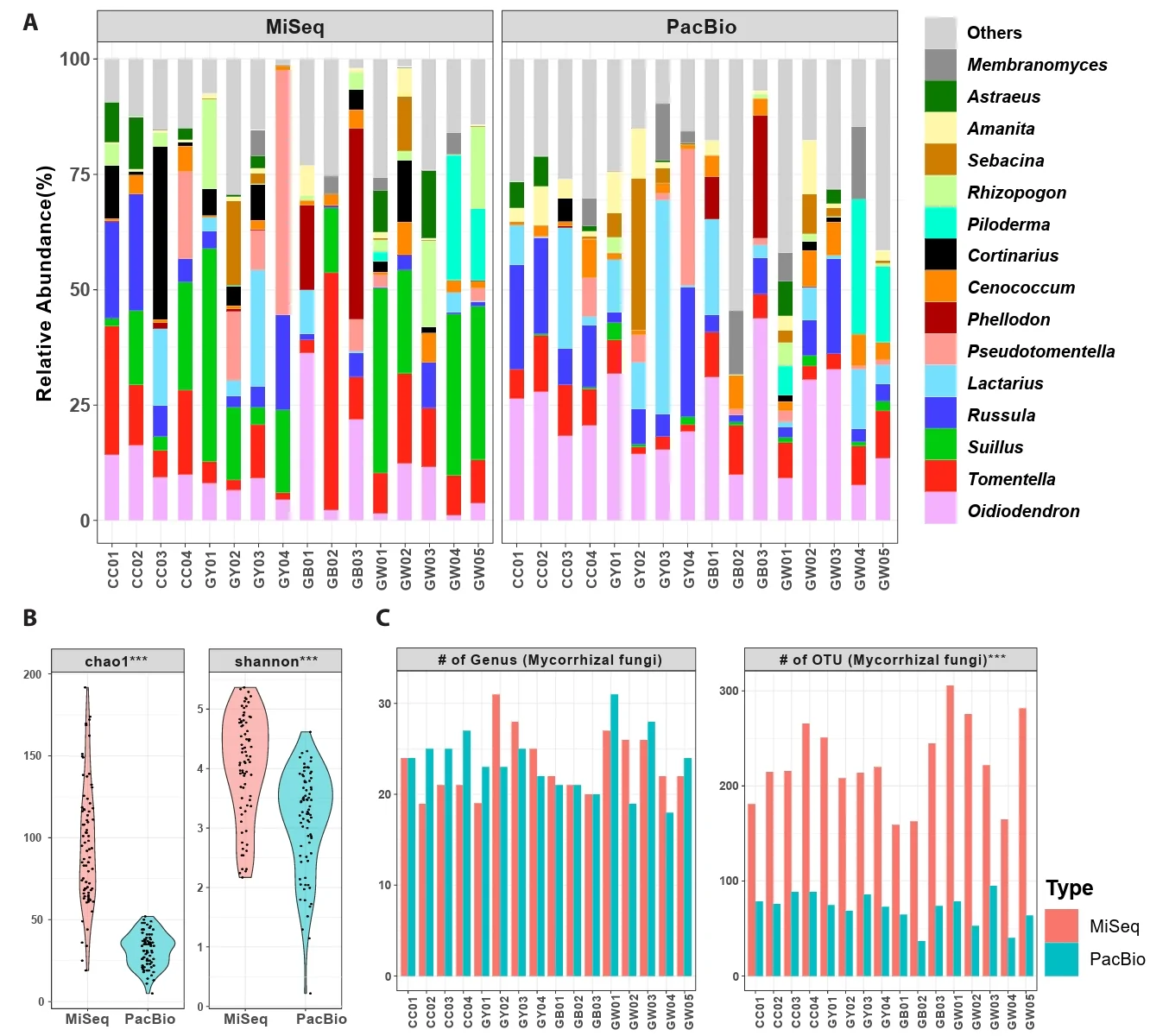
Comparison of mycorrhizal diversity and composition between MiSeq (ITS2) and PacBio (full ITS) datasets. (A) Relative abundance of the top 15 mycorrhizal genera in each sampling site. The order of genera was assigned according to the average relative abundance of MiSeq and PacBio datasets. (B) Diversity indices (Chao1 richness and Shannon-Wiener diversity) of root-associated mycorrhizal fungi in *P. densiflora* (paired t-test). (C) The number of mycorrhizal genera and OTUs found in each sampling sites. Red bars indicate MiSeq datasets, and blue bars indicate PacBio datasets. Data are shown as mean relative abundance per forest, calculated from five root samples (one per tree) collected in each forest (n = 16 forests; 80 samples in total). For each group, significant differences between datasets were marked with * (paired t-test, *p ≤ 0.05, **p ≤ 0.01, ***p ≤ 0.001).

Differences in mycorrhizal diversity were analyzed between the two datasets. Despite using the same samples, the abundance of fungal taxa was highly different between datasets (Fig. 2A). The composition greatly differed at the genus level (Jaccard: R^2^ = 0.10, p = 0.001; Bray-Curtis: R^2^ = 0.14, p = 0.001), although Procrustes analysis revealed significant correlations in both genus (Jaccard: M^2^ = 0.52, correlation = 0.68, p = 0.0008; Bray-Curtis: M^2^ = 0.54, correlation = 0.67, p = 0.0008) and OTU levels (M^2^ = 0.66, correlation = 0.58, p = 0.0077; Bray-Curtis: M^2^ = 0.70, correlation = 0.55, p = 0.015). Similarly, mantel test showed significant correlations at the genus level (Bray-Curtis: R = 0.504, p = 0.003, Jaccard: R = 0.504, p = 0.001), and at the OTU level (Bray-Curtis: R = 0.796, p = 0.001, Jaccard: R = 0.796, p = 0.001). For alpha diversity, richness and diversity indices were significantly higher in the MiSeq dataset than in the PacBio dataset (Fig. 2B). This difference was explained by variation in the number of OTUs per sample rather than by the number of genera (Fig. 2C). A significant positive correlation was detected between the two datasets (R^2^ = 0.38, p < 0.001).

### Local database inclusion effect

**Fig. 3.**
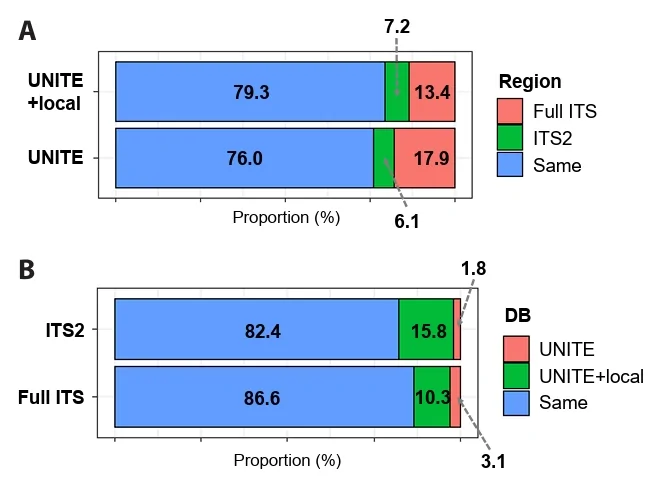
Effect of marker region (full ITS and ITS2-extracted) and database on identification resolution in the PacBio datasets. (A) Improvement in identification resolution due to marker region selection. (B) Improvement in identification resolution due to database selection. Proportion (%) was measured based on the number of OTUs, and identification resolution was based on taxonomic ranks (i.e. species > genus > family).

To determine the influence of including local databases on identification resolution, representative sequences from PacBio sequencing were analyzed in two datasets (PacBio Full ITS and PacBio ITS2-extracted), and taxonomic assignment was performed using the UNITE and UNITE+local databases (Fig. 3). Regardless of the database used, the full ITS region (UNITE+local: 13.4% and UNITE: 17.9%) obtained better identification resolution (i.e. lower taxonomic rank) than the ITS2 region (UNITE+local: 7.2% and UNITE: 6.1%) (Fig. 3A). Class-level comparison further revealed that, regardless of the database used, the use of the full-length ITS region improved identification resolution in specific fungal lineages (e.g., Leotiomycetes, Saccharomycetes, and Mortierellomycetes) compared with the ITS2-extracted dataset (Fig. S3). Regarding the influence of the reference database, supplementing it with local sequences yielded higher resolution irrespective of the marker region (Fig. 3B) and the improvement was more pronounced in the shorter ITS2 region than in the full ITS region, as reflected by the higher proportion of identified sequences (ITS2: 15.8% and Full ITS: 10.3%). In addition, class-level analyses showed that, regardless of marker region length, the inclusion of the local database enhanced identification resolution in several specific lineages (e.g., Agaricomycetes, Leotiomycetes, Tremellomycetes, and Mortierellomycetes) (Fig. S4).

## Discussion

This study provides a comprehensive comparison of two NGS platforms (Illumina MiSeq and PacBio Sequel) using data from the root-associated mycorrhizal mycobiome of *Pinus densiflora*. By assessing taxonomic assignment and diversity between short- (MiSeq) and long-read (PacBio) sequencing data, we demonstrate how methodological choices can affect the interpretation of community composition. Furthermore, we tested the importance of supplementing global databases with local reference sequences to improve taxonomic resolution.

For community composition and diversity analyses, the Miseq and PacBio platforms captured a substantial overlap in community composition, accounting for 95% at the genus level. At the species level, 44 species were shared between the datasets. These shared species represented more than half of the total sequence abundance, indicating a core mycobiome consistently detected across methods. Notable genera include *Lactarius*, *Oidiodendron*, *Russula*, *Suillus*, *Cenococcum*, and *Tomentella*, which have been repeatedly associated with *P. densiflora* (Lee and Eom, 2013; Toju and Sato, 2018) as well as with other *Pinus* species worldwide (Barroetaveña et al., 2007; Bowman et al., 2021; Mandolini et al., 2022). Beyond this overlap, more taxa were detected exclusively in the PacBio dataset (37) than in the Miseq dataset (17), particularly among those with low sequence abundance. This highlights the advantage of full-length ITS sequencing in detecting rare or closely related lineages with higher resolution (Tedersoo et al., 2022).

Nevertheless, MiSeq yielded a greater number of mycorrhizal OTUs, which likely reflects its deeper sequencing depth rather than inherently higher diversity, as indicated by the significant correlation in OTU counts between platforms. Some genera, such as *Suillus*, *Cortinarius*, *Inocybe*, and *Tricholoma*, appeared at relatively higher frequencies in the MiSeq dataset. This discrepancy may be attributable to differences in primer sets used for each platform, as primer choice can significantly influence the detected fungal community structure (Tedersoo et al., 2018). Although both primer sets exhibit high overall coverage across fungal lineages (Nilsson et al., 2019), minor lineage-specific amplification bias cannot be completely excluded and may contribute to subtle differences in detected community composition. Further research is needed to determine whether such patterns are consistently reproducible across studies.

The two platforms differed in their capacity for taxonomic resolution. Consistent with previous studies (Tedersoo et al., 2018; Walder et al., 2017), full-length ITS data (PacBio) generally outperformed ITS2 data (MiSeq) in species level resolution. Due to shorter sequence reads, the MiSeq dataset yielded lower resolution at the species level (Nilsson et al., 2019) compared to the full-length ITS reads obtained from the PacBio dataset. Additional analyses of ITS2 and full-length ITS datasets derived from PacBio sequencing further supported the importance of sequence length in taxon identification at a finer resolution.

Taxonomic assignment success increased when local reference sequences were added to the global database, a trend especially evident in the ITS2 dataset. This result indicates the important role of customized reference datasets in mycobiome studies (Nilsson et al., 2019). In particular, local databases not only reduce the impact of taxonomic bias in global repositories but also reflect the regional uniqueness of fungal assemblages (Větrovský et al., 2020). Notably, recent phylogenetic studies have shown that many Asian mycorrhizal fungi differ from morphologically similar taxa described from Europe or North America (Cho et al., 2018; Lee et al., 2017, 2019, 2021; Wisitrassameewong et al., 2020). For example, several *Lactarius* species identified in this study (e.g., *L. betulinus*, *L. citrinus*, *L. lutescens*) were previously documented through specimen-based studies in Korea as distinct from morphologically similar species elsewhere (Lee et al., 2019). Incorporating sequences from well-identified local specimens can therefore significantly enhance molecular identification in metabarcoding applications.

Our results have two main implications for mycorrhizal community studies. First, long-read sequencing (PacBio) is advantageous for resolving rare or closely related taxa because the full-length ITS provides extended sequence information that can also match partial ITS regions in reference databases (Tedersoo et al., 2021). Similar findings were reported in a mycobiome study comparing Oxford Nanopore Technologies (ONT) and Illumina MiSeq, where ONT detected more unique genera than MiSeq (Mittelstrass et al., 2025). Supplementing databases with local sequence data further increases the detection of unique taxa. In contrast, short-read sequencing (MiSeq) offers higher sequencing depth, which increases OTU recovery and enhances the detection of more abundant community members. Second, primer selection can introduce biases that influence the relative detection of certain taxa, as observed with *Suillus* and *Rhizopogon* in MiSeq versus *Amanita* in PacBio. Using multiple primer pairs may broaden coverage, but the risk of unintended primer interactions must be carefully considered (Tedersoo et al., 2022).

In conclusion, the two sequencing platforms offer complementary strengths for studying mycorrhizal communities: MiSeq remains cost-efficient and powerful for large-scale community screening, while PacBio enhances taxonomic resolution for rare and closely related taxa. Maximizing the accuracy of mycorrhizal community profiling will require careful consideration of sequencing depth, marker region, and primer selection, as well as the use of curated local reference databases. Our findings provide methodological guidance for future metabarcoding studies and highlight the importance of selecting sequencing strategies that align with specific ecological and taxonomic objectives.

## Supplementary Information

Fig. S1. The rarefaction curve for PacBio (full ITS) and MiSeq (ITS2) datasets.

Fig. S2. Length distribution of MiSeq and PacBio OTUs. Histograms show the distribution of OTU sequence lengths obtained from Illumina MiSeq (ITS2 region) and PacBio (full-length ITS) datasets. Each dashed vertical line indicates the mean sequence length for each platform.

Fig. S3. Class-level details of the improvement in identification resolution associated with marker region selection (length effect). "_un" indicates unclassified taxa. The horizontal lines below the x-axis group classes belonging to the same phylum, with abbreviated phylum names shown: As, Ascomycota; Ba, Basidiomycota; Mo, Mortierellomycota; Mu, Mucoromycota, Ro, Rozellomycota.

Fig. S4. Class-level details of the improvement in identification resolution associated with database selection (DB effect). "_un" indicates unclassified taxa. The horizontal lines below the x-axis group classes belonging to the same phylum, with abbreviated phylum names shown: As, Ascomycota; Ba, Basidiomycota; Mo, Mortierellomycota; Mu, Mucoromycota, Ro, Rozellomycota.

Table S1. Metadata of 80 root samples used in this study

Table S2. Taxonomic summary of species number in the SFC database. The number of unique species is compared with the UNITE database (v. 8.2).

## References

[b1-jm-2509008] Abarenkov K, Nilsson RH, Larsson KH, Alexander IJ, Eberhardt U (2010). The UNITE database for molecular identification of fungi-recent updates and future perspectives. New Phytol.

[b2-jm-2509008] Barroetaveña C, Cázares E, Rajchenberg M (2007). Ectomycorrhizal fungi associated with ponderosa pine and Douglas-fir: a comparison of species richness in native western North American forests and Patagonian plantations from Argentina. Mycorrhiza.

[b3-jm-2509008] Bengtsson-Palme J, Ryberg M, Hartmann M, Branco S, Wang Z (2013). Improved software detection and extraction of ITS1 and ITS2 from ribosomal ITS sequences of fungi and other eukaryotes for analysis of environmental sequencing data. Methods Ecol Evol.

[b4-jm-2509008] Bokulich NA, Subramanian S, Faith JJ, Gevers D, Gordon JI (2013). Quality-filtering vastly improves diversity estimates from Illumina amplicon sequencing. Nat Methods.

[b5-jm-2509008] Bowman EA, Hayden DR, Arnold AE (2021). Fire and local factors shape ectomycorrhizal fungal communities associated with *Pinus ponderosa* in mountains of the Madrean Sky Island Archipelago. Fungal Ecol.

[b6-jm-2509008] Brundrett MC (2009). Mycorrhizal associations and other means of nutrition of vascular plants: understanding the global diversity of host plants by resolving conflicting information and developing reliable means of diagnosis. Plant Soil.

[b7-jm-2509008] Caporaso JG, Kuczynski J, Stombaugh J, Bittinger K, Bushman FD (2010). QIIME allows analysis of high-throughput community sequencing data. Nat Methods.

[b8-jm-2509008] Castaño C, Berlin A, Brandström Durling M, Ihrmark K, Lindahl BD (2020). Optimized metabarcoding with Pacific biosciences enables semi-quantitative analysis of fungal communities. New Phytol.

[b9-jm-2509008] Cho HJ, Park MS, Lee H, Oh SY, Wilson AW (2018). A systematic revision of the ectomycorrhizal genus *Laccaria* from Korea. Mycologia.

[b10-jm-2509008] Cho Y, Yoo S, Park MS, Kim JS, Kim CS, Lim YW (2021). Ectomycorrhizal fungi associated with *Pinus densiflora* seedlings under flooding stress. Sustainability.

[b11-jm-2509008] Choung Y, Lee J, Cho S, Noh J (2020). Review on the succession process of *Pinus densiflora* forests in South Korea: Progressive and disturbance-driven succession. J Ecol Environ.

[b12-jm-2509008] Frøslev TG, Kjøller R, Bruun HH, Ejrnæs R, Brunbjerg AK (2017). Algorithm for post-clustering curation of DNA amplicon data yields reliable biodiversity estimates. Nat Commun.

[b13-jm-2509008] Furneaux B, Bahram M, Rosling A, Yorou NS, Ryberg M (2021). Long- and short-read metabarcoding technologies reveal similar spatiotemporal structures in fungal communities. Mol Ecol Resour.

[b14-jm-2509008] Gower JC (1975). Generalized procrustes analysis. Psychometrika.

[b15-jm-2509008] Hu Y, Irinyi L, Hoang MTV, Eenjes T, Graetz A (2022). Inferring species compositions of complex fungal communities from long- and short-read sequence data. mBio.

[b16-jm-2509008] Kennedy PG, Cline LC, Song Z (2018). Probing promise versus performance in longer read fungal metabarcoding. New Phytol.

[b17-jm-2509008] Lee EH, Eom AH (2013). Ectomycorrhizal fungal communities of red pine (*Pinus densiflora*) seedlings in disturbed sites and undisturbed old forest sites. Mycobiology.

[b18-jm-2509008] Lee H, Park MS, Jung PE, Eimes JA, Seok SJ (2017). Re-evaluation of the taxonomy and diversity of *Russula* section *Foetentinae* (Russulales, Basidiomycota) in Korea. Mycoscience.

[b19-jm-2509008] Lee H, Wissitrassameewong K, Park MS, Fong JJ, Verbeken A (2021). Taxonomic revision of the genus *Lactifluus* (Russulales, Basidiomycota) of South Korea. Mycobiology.

[b20-jm-2509008] Lee H, Wissitrassameewong K, Park MS, Verbeken A, Eimes J (2019). Taxonomic revision of the genus *Lactarius* (Russulales, Basidiomycota) in Korea. Fungal Divers.

[b21-jm-2509008] Mandolini E, Bacher M, Peintner U (2022). Ectomycorrhizal fungal communities of Swiss stone pine (*Pinus cembra*) depend on climate and tree age in natural forests of the Alps. Plant Soil.

[b22-jm-2509008] Mittelstrass J, Heinzelmann R, Eschen R, Hartmann M, Kupper Q (2025). Metabarcoding with Illumina and Oxford Nanopore Technologies provides complementary insights into tree seed mycobiota. Environ Microbiome.

[b23-jm-2509008] Nguyen NH, Song Z, Bates ST, Branco S, Tedersoo L (2016). FUNGuild: an open annotation tool for parsing fungal community datasets by ecological guild. Fungal Ecol.

[b24-jm-2509008] Nilsson RH, Anslan S, Bahram M, Wurzbacher C, Baldrian P (2019). Mycobiome diversity: high-throughput sequencing and identification of fungi. Nat Rev Microbiol.

[b25-jm-2509008] https://cran.r-project.org/web/packages/vegan.

[b26-jm-2509008] Park KH, Oh SY, Yoo S, Park MS, Fong JJ (2020). Successional change of the fungal microbiome pine seedling roots Inoculated with *Tricholoma matsutake*. Front Microbiol.

[b27-jm-2509008] Park KH, Yoo S, Park MS, Kim CS, Lim YW (2021). Different patterns of belowground fungal diversity along altitudinal gradients with respect to microhabitat and guild types. Environ Microbiol Rep.

[b28-jm-2509008] Policelli N, Horton TR, Hudon AT, Patterson TR, Bhatnagar JM (2020). Back to roots: the role of ectomycorrhizal fungi in boreal and temperate forest restoration. Front For Glob Change.

[b29-jm-2509008] Põlme S, Abarenkov K, Nilsson RH, Lindahl BD, Clemmensen KE (2020). FungalTraits: a user-friendly traits database of fungi and fungus-like stramenopiles. Fungal Divers.

[b30-jm-2509008] https://www.R-project.org.

[b31-jm-2509008] Rognes T, Flouri T, Nichols B, Quince C, Mahé F (2016). VSEARCH: a versatile open source tool for metagenomics. PeerJ.

[b32-jm-2509008] Ruppert KM, Kline RJ, Rahman MS (2019). Past, present, and future perspectives of environmental DNA (eDNA) metabarcoding: A systematic review in methods, monitoring, and applications of global eDNA. Glob Ecol Conserv.

[b33-jm-2509008] Sim MY, Eom AH (2009). Diversity of ectomycorrhizal fungi of *Pinus densiflora* Siebold et Zucc. seedlings in a disturbed forest on Mt. Songni. J Ecol Field Biol.

[b35-jm-2509008] Taylor DL, McCormick MK (2008). Internal transcribed spacer primers and sequences for improved characterization of basidiomycetous orchid mycorrhizas. New Phytol.

[b36-jm-2509008] Tedersoo L, Albertsen M, Anslan S, Callahan BJ (2021). Perspectives and Benefits of High-Throughput Long-Read Sequencing in Microbial Ecology. Appl Environ Microbiol.

[b37-jm-2509008] Tedersoo L, Bahram M, Zinger L, Nilsson RH, Kennedy PG (2022). Best practices in metabarcoding of fungi: from experimental design to results. Mol Ecol.

[b38-jm-2509008] Tedersoo L, Jairus T, Horton BM, Abarenkov K, Suvi T (2008). Strong host preference of ectomycorrhizal fungi in a Tasmanian wet sclerophyll forest as revealed by DNA barcoding and taxon-specific primers. New Phytol.

[b39-jm-2509008] Tedersoo L, Tooming-Klunderud A, Anslan S (2018). PacBio metabarcoding of Fungi and other eukaryotes: errors, biases and perspectives. New Phytol.

[b40-jm-2509008] Toju H, Sato H (2018). Root-associated fungi shared between arbuscular mycorrhizal and ectomycorrhizal conifers in a temperate forest. Front Microbiol.

[b41-jm-2509008] Větrovský T, Morais D, Kohout P, Lepinay C, Algora C (2020). GlobalFungi, a global database of fungal occurrences from high-throughput-sequencing metabarcoding studies. Sci Data.

[b42-jm-2509008] Walder F, Schlaeppi K, Wittwer R, Held AY, Vogelgsang S (2017). Community profiling of *Fusarium* in combination with other plant-associated fungi in different crop species using SMRT sequencing. Front Plant Sci.

[b43-jm-2509008] Wisitrassameewong K, Park MS, Lee H, Ghosh A, Das K (2020). Taxonomic revision of *Russula* subsection *Amoeninae* from South Korea. MycoKeys.

[b44-jm-2509008] Yoo S, Cho Y, Park KH, Lim YW (2022). Exploring fine-scale assembly of ectomycorrhizal fungal communities through phylogenetic and spatial distribution analyses. Mycorrhiza.

