# Supplementary material for: Effects of sequencing platforms on the profiling of root mycorrhizal communities in Pinus densiflora
Source: J Microbiol. 2026 Jan 31;64(1):e2509008. doi: 10.71150/jm.2509008 (PMC13577058; doi:10.71150/jm.2509008)
Supplement: Supplementary file 1 [file jm-2509008-Supplementary-Fig-S1.pdf]

Supplementary data

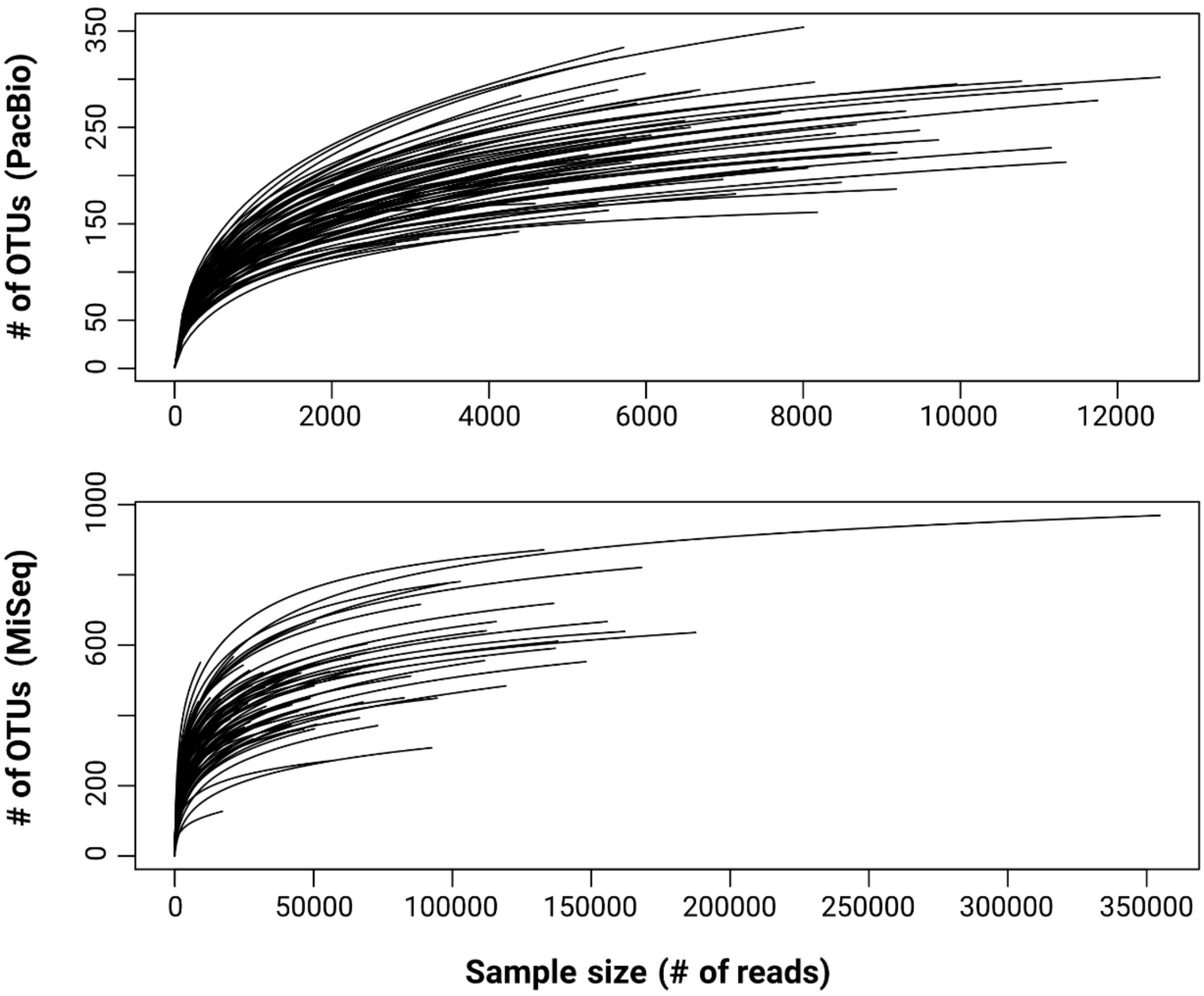

Fig. S1. The rarefaction curve for PacBio (full ITS) and MiSeq (ITS2) datasets.
