# Supplementary material for: Effects of sequencing platforms on the profiling of root mycorrhizal communities in Pinus densiflora
Source: J Microbiol. 2026 Jan 31;64(1):e2509008. doi: 10.71150/jm.2509008 (PMC13577058; doi:10.71150/jm.2509008)
Supplement: Supplementary file 2 [file jm-2509008-Supplementary-Fig-S2.pdf]

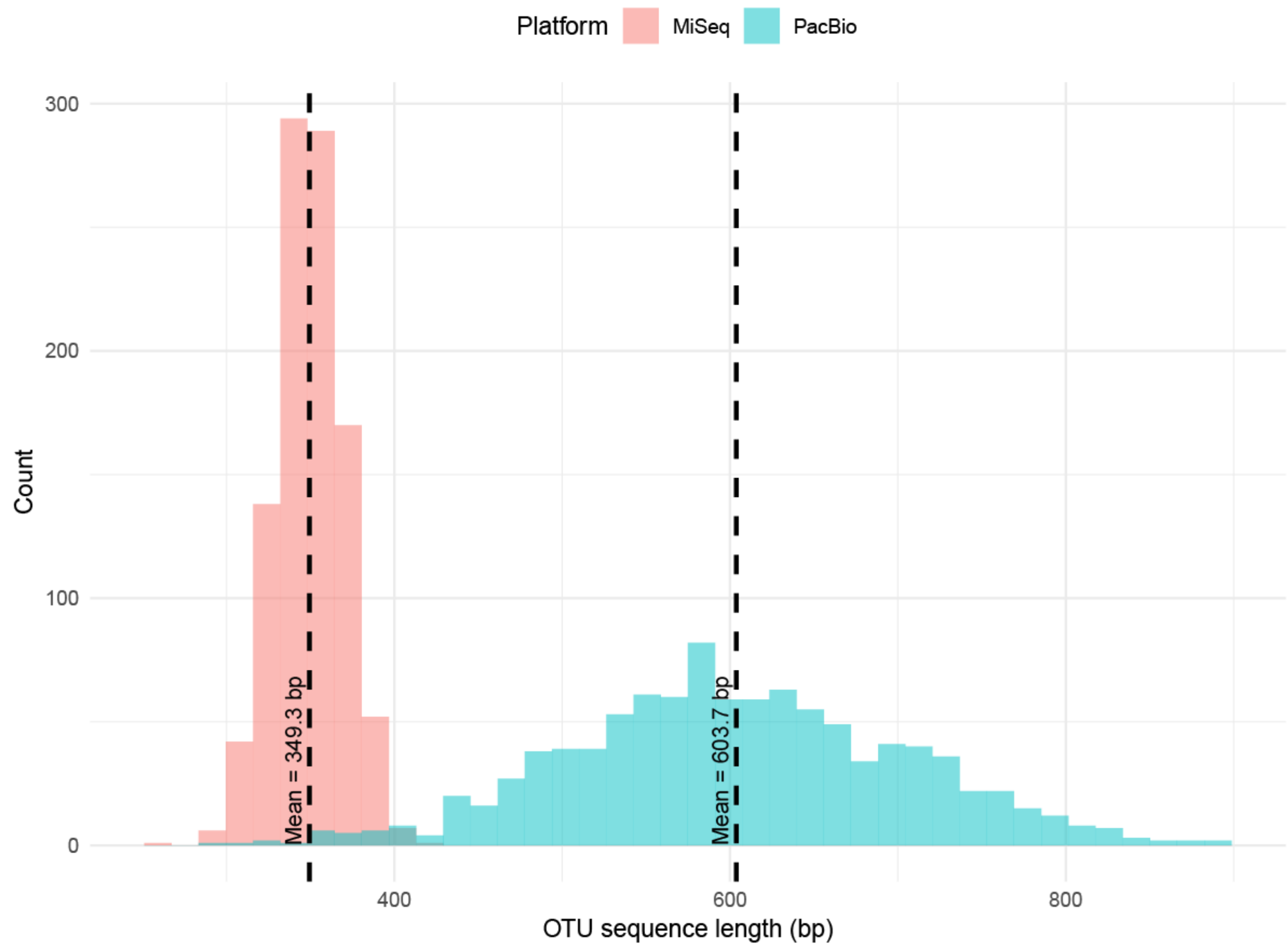

Fig. S2. Length distribution of MiSeq and PacBio OTUs. Histograms show the distribution of OTU sequence lengths obtained from Illumina MiSeq (ITS2 region) and PacBio (full-length ITS) datasets. Each dashed vertical line indicates the mean sequence length for each platform.
