# Supplementary material for: Effects of sequencing platforms on the profiling of root mycorrhizal communities in Pinus densiflora
Source: J Microbiol. 2026 Jan 31;64(1):e2509008. doi: 10.71150/jm.2509008 (PMC13577058; doi:10.71150/jm.2509008)
Supplement: Supplementary file 3 [file jm-2509008-Supplementary-Fig-S3.pdf]

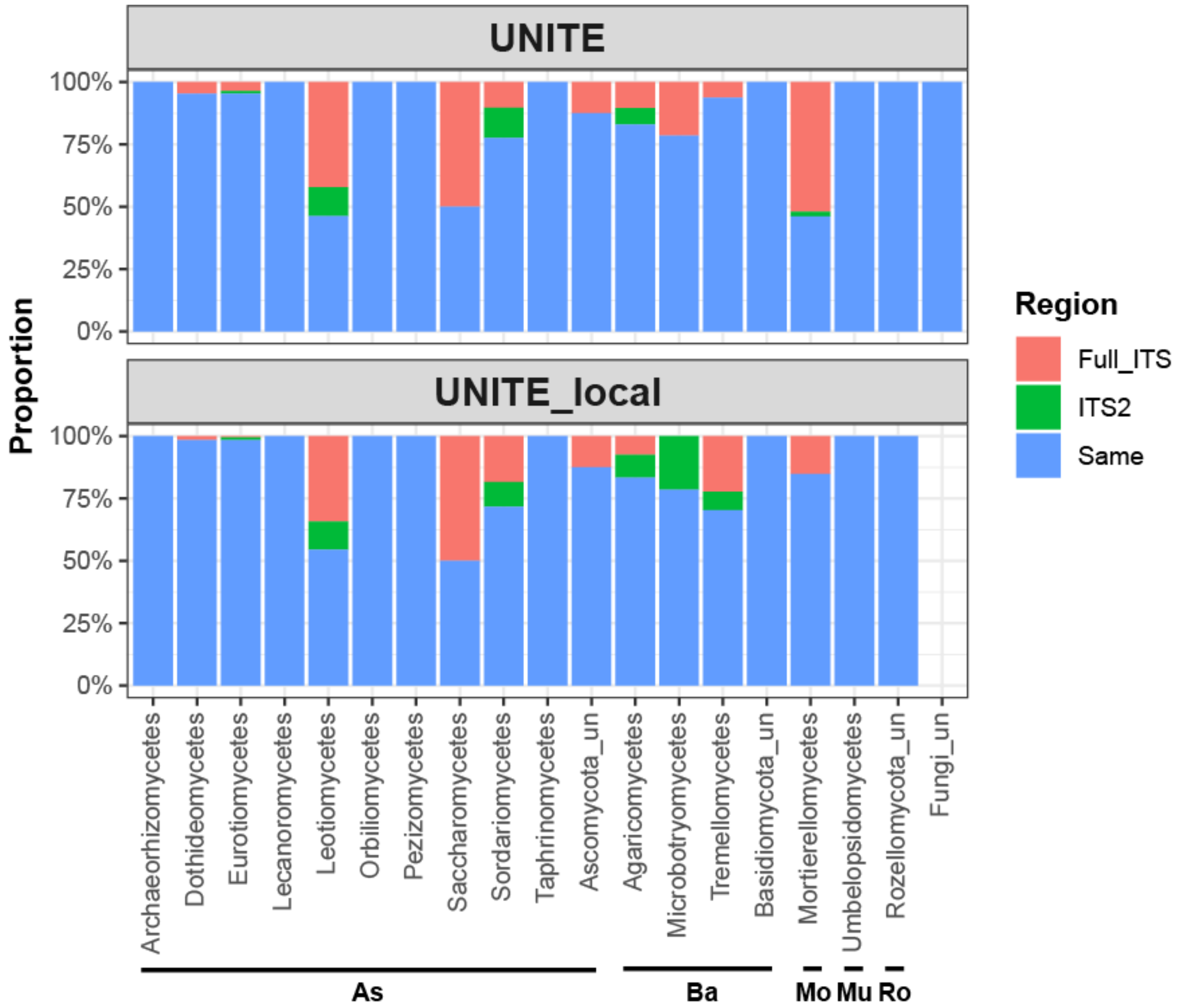

Fig. S3. Class-level details of the improvement in identification resolution associated with marker region selection (length effect). "_un" indicates unclassified taxa. The horizontal lines below the x-axis group classes belonging to the same phylum, with abbreviated phylum names shown: As, Ascomycota; Ba, Basidiomycota; Mo, Mortierellomycota; Mu, Mucoromycota, Ro, Rozellomycota.
